# Research advances in the understanding of how exosomes regulate ferroptosis in cancer

**DOI:** 10.1007/s12094-023-03089-6

**Published:** 2023-01-27

**Authors:** Jiaxuan Liu

**Affiliations:** The School of Public Health in Qingdao University, Qingdao, China

**Keywords:** Exosome, Ferroptosis, Cancer

## Abstract

Exosomes are extracellular vesicles that can release different bioactive substances to affect tumor cells and cell death pathways. As an important mediator of cell communication, exosomes participate in the occurrence and development of a variety of diseases. Ferroptosis, one of the newly defined forms of regulated cell death, is characterized by massive accumulation of iron ions and lipid peroxidation. An increasing number of studies have shown that ferroptosis plays an important role in malignant tumors. Moreover, exosomes have been recognized for their potential in cancer therapy based on ferroptosis. To further describe how could exosomes regulate ferroptosis in cancer and provide better understanding of the mechanisms involved, this paper reviews the definition as well as the underlying molecular mechanisms of ferroptosis, including iron metabolism, amino acid metabolism, lipid metabolism and so on. Then, we illustrated how could exosomes regulate the ferroptosis pathway and suggested their promising potential as a novel tumor therapy for cancer patients. Finally, we described the perspectives of ferroptosis by exosomes in tumor treatment. Therefore, exosomes have the potential to regulate ferroptosis in clinical cancer treatment.

## Introduction

Exosomes are specific extracellular vesicles (EVs) released into the extracellular environment after the fusion of intracellular multivesicular bodies (MVBs) and living cell membranes; these EVs have a bilayer membrane structure with a diameter of 40–160 nm [1]. The production of exosomes involves double invagination of the plasma membrane and the formation of MVBs containing intraluminal vesicles (ILVs), which are secreted in the form of exosomes through the fusion and endocytosis of MVBs and plasmalemma [2]. Exosomes, as an important mediator of intercellular communication, participate in physiological and pathological processes in many diseases. Studies have shown that thrombospondin 5 released from insulin-resistant adipocyte-derived exosomes can induce the migration and invasion of breast cancer cells [3]. Some studies have also shown that bone marrow mesenchymal stem cell-derived exosomes (BM-MSC-exos) can upregulate the expression of S100A4 to promote the proliferation of acute myeloid leukemia (AML) cells and enhance the resistance of AML cells to cytarabine [4]. In contrast, the release of miR-186 from natural killer (NK) cell-derived exosomes can inhibit the growth of neuroblastoma cells [5]. These results show that exosomes from different sources can have unique effects on tumor cells. In recent years, research regarding mechanisms by which exosomes regulate tumor cell ferroptosis has attracted the attention of researchers. The relationship between exosomes and ferroptosis urgently needs to be explored, and new treatment strategies for cancer are needed.

## Ferroptosis

Ferroptosis is a novel type of programmed cell death that depends on the massive accumulation of iron and lipid peroxidation [6]. It features a reduction in size or disappearance of the mitochondrial crest, rupture of the mitochondrial outer membrane, an increase in membrane density, preservation of the structural integrity of the nucleus, and loss of the chromatin edge, among other changes [7]. The underlying molecular mechanisms of ferroptosis are closely related to iron metabolism, amino acid metabolism, lipid metabolism and other metabolism.

### Iron metabolism

Iron metabolism-related proteins such as transferrin (TRF) and transferrin receptor 1 (TFR1) are the key mediators of ferroptosis. The transport of iron from extracellular environment to cells by TRF and TFR1 is a necessary step in ferroptosis. The research found that extracellular Fe^3+^ enters the endosome through TFR1, and then Fe^3+^ is reduced to Fe^2+^, which increases the level of intracellular iron and induces ferroptosis [8]. Tang et al. found that ubiquitin-specific protease 7 (USP7) can increase iron uptake and promote ferroptosis by activating p53/TFR1 pathway in rat hearts after ischemia/reperfusion [9]. Jiang et al. found that long-term hyperinsulinemia increases liver TFR1 through PI3K/IRP2 pathway, leading to the liver iron overload, which may be one of the main causes of metabolic abnormal iron overload syndrome [10]. Yang et al. found that autophagy can selectively degrade the core circadian clock protein aryl hydrocarbon receptor nuclear translocator-like protein 1 (ARNTL), and ARNTL inhibit the transcription of egl nine homolog 2 (EGLN2), which mediates the downregulation of hypoxia-inducible factor 1 subunit α (HIF1A) to promote ferroptosis [11]. Hou et al. found that knockout of autophagy-related gene 5 (ATG5) and autophagy-related gene 7 (ATG7) can inhibit erastin-induced ferroptosis by reducing intracellular ferrous level and lipid peroxidation [12]. Meanwhile, iron autophagy is a selective autophagy process in which lysosomes degrade intracellular ferritin, release free iron and cause oxidative damage mediated by nuclear receptor coactivator 4 (NCOA4). Inhibition of lysosome function or silencing NCOA4 can inhibit ferroptosis in tumor cells [13]. Turcu et al. found that divalent metal transporter 1 (DMT1) inhibitors selectively target cancer stem cells by blocking lysosomal iron transport, resulting in the accumulation of lysosomal iron, the production of reactive oxygen species (ROS) and ferroptosis. Therefore, the input, discharge, storage and flow rate of iron will affect the occurrence of ferroptosis.

### Amino acid metabolism

GSH, as the substrate of glutathione peroxidase 4 (GPX4), participates in intracellular antioxidant response, which is a key factor in ferroptosis. Cystine/glutamate transporters (also called system XC-) receptor is closely related to ferroptosis, which converts intracellular glutamate to cystine at 1:1, which is reduced to cysteine after entering the cell, and then synthesizes GSH to regulate downstream lipid peroxidation [14]. Inhibition of system XC-metabolism can lead to an imbalance in amino acid metabolism and trigger ferroptosis. Glutamine is also an important factor in mediating ferroptosis. When glutamine is insufficient or its decomposition is blocked, it cannot induce the accumulation of ROS, which leads to lipid peroxidation and ferroptosis [14].

### Lipid metabolism

Lipid oxidation stress and its membrane damage are considered to be the landmark events of ferroptosis, especially polyunsaturated fatty acids (PUFAs) are more likely to form lipid peroxides and trigger ferroptosis. At present, it is believed that the accumulation of PUFA is the sign of ferroptosis, and the content of intracellular PUFA determines the development degree of ferroptosis [15]. The accumulation of PUFA is a complex process. Inhibition of GPX4 will lead to the accumulation of PUFA and ROS, damage of plasma membrane integrity and ferroptosis. The substrate of lipid peroxidation is fatty acid, which includes PUFA and monounsaturated fatty acid (MUFA), while PUFA is more likely to be oxidized than MUFA [16]. Therefore, reducing MUFA content and increasing PUFA content can promote the progress of ferroptosis induced by lipid peroxidation. In addition, there are three key enzymes in the enzymatic reaction of lipid peroxidation: long-chain acyl-CoA synthetase 4 (ACSL4), Lysophosphatidylcholine Acyltransferase 3 (LPCAT3) and arachidonate lipoxygenase 15 (ALOX15). Among them, ACSL4 was involved in the catalytic esterification of free PUFAs and was incorporated into membrane phospholipids with the help of LPCAT3. Subsequently, ALOX15 participates in the peroxidation of membrane phospholipids [17]. Therefore, ACSL4/LPCTA3/ALOX15 pathway can promote ferroptosis induced by lipid peroxidation.

### Other metabolism

**Fig. 1:**
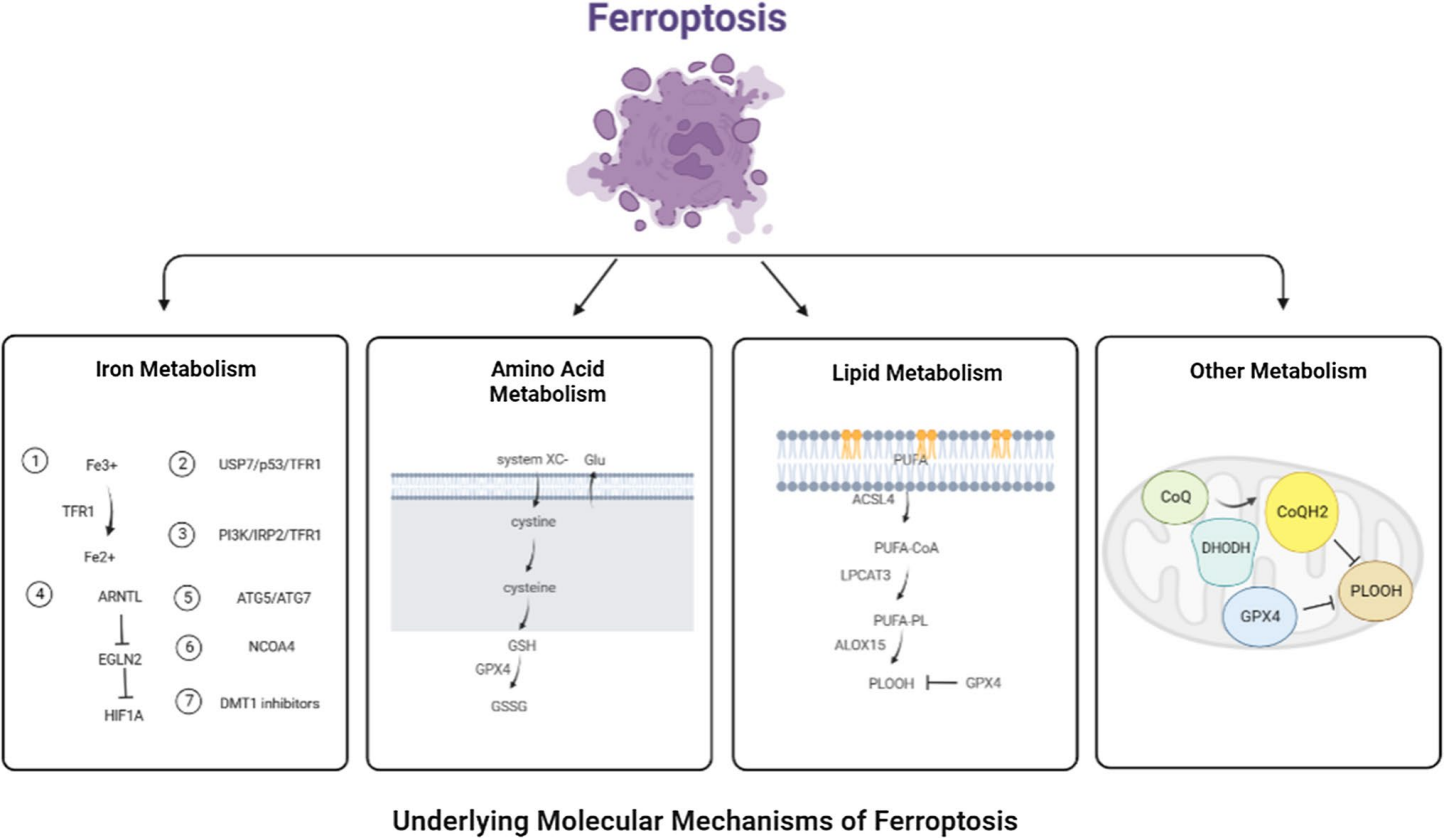
Underlying molecular mechanisms of ferroptosis. The underlying molecular mechanisms of ferroptosis is closely related to iron metabolism, amino acid metabolism, lipid metabolism and other metabolism

Dihydroorotate Dehydrogenase (DHODH) is a Flavin-dependent enzyme located in the inner membrane of mitochondria, which can oxidize dihydrowhey acid to whey acid and provide electrons to CoQ so that it can be reduced to CoQH2 and inhibit the progress of ferroptosis [18]. Mao et al. found that the addition of DHODH inhibitor BQR to the cell lines with low expression of GPX4 could induce ferroptosis, while the addition of BQR to the cells with high expression of GPX4 could increase the sensitivity of cells to ferroptosis inducers. Therefore, DHODH is a ferroptosis inhibitor independent of GPX4 pathway, and inhibiting the expression of DHODH may be a new strategy to induce ferroptosis [19] (Fig. 1).

Ferroptosis can be induced by extrinsic or intrinsic pathways, and the extrinsic pathway is initiated by inhibiting cell membrane transporters, such as system XC-, or activating iron transporters, such as transferrin and lactotransferrin. The intrinsic pathway is activated by blocking intracellular antioxidant enzymes such as GPX4 [20]. Although it is known that this process does not involve the activity of caspases, MLKL or gasdermin D, the effector molecules of ferroptosis remains to be identified [21]. In recent years, substantial progress has been made in understanding the role of ferroptosis in tumor biology and cancer treatment. Early preclinical studies have found links between some carcinogenic signals and ferroptosis: (1) the ferroptosis activator erastin selectively triggers the death of RAS-mutant tumor cells but not wild-type-RAS tumor cells [22]. (2) Erastin-induced cell death requires activation of the RAS-RAF-MEK-ERK pathway [23]. (3) Iron is not only important for tumor cell proliferation but also necessary for erastin-induced cell death [24]. Subsequent studies have determined that ferroptosis is controlled through complex signaling pathways related to iron accumulation, lipid peroxidation and membrane damage. Furthermore, tumor cells that are resistant to traditional therapy or have a high tendency to metastasize may be particularly vulnerable to ferroptosis, thus revealing the potential of inducing ferroptosis as targeted therapy [25, 26]. As a supplement to the previous review of exosomes and ferroptosis [27, 28], here, we focus on the mechanisms by which exosomes regulate ferroptosis, the regulatory effects by which exosomes promote or inhibit ferroptosis, and the application of both types of effects in tumor therapy.

## Exosomes regulate the ferroptosis pathway

### Ferritin metabolism

One study found that prominin-2 is a ferroptosis stress response protein that promotes the formation of ferritin-containing MVBs and exosomes, which transport iron out of cells, thereby inhibiting ferroptosis in breast epithelium and breast cancer cell ferroptosis, blocking ferritin output mediated by MVBs and exosomes, and inhibiting the expression of GPX4, all of these effects result in intracellular iron accumulation [29]. That study proposed for the first time that exosome scavenging of intracellular iron ions is an important mechanism driving ferroptosis resistance, and this finding is of broad significance for further revealing the relationship between exosomes and ferroptosis. Endothelial exosomes can reverse glucocorticoid-induced osteoblast inhibition by inhibiting ferritin autophagy-dependent ferroptosis [30]. In addition, ferroptosis-dependent EVs secreted by macrophages promote the occurrence of asbestos-related mesothelioma by loading ferritin [31]. Therefore, exosomes containing ferritin play an important role in regulating iron metabolism and affecting the sensitivity to ferroptosis.

### Lipid metabolism

Zhang et al. found that the secretion of miR-522 from cancer-associated fibroblast-derived exosomes (CAF-exos) inhibits the accumulation of lipid peroxides by inhibiting the expression of ALOX15, thus inhibiting the occurrence of ferroptosis in gastric cancer (GC) cells [32]. That study proved for the first time that exosomes are related to lipid peroxidation in ferroptosis. Another study proved that exosomes released by tumor cells can specifically reduce the level of lipid peroxidation, reduce the formation of ROS, inhibit ferroptosis and block exosome biosynthesis by increasing the expression of circRNA_101093 (cir93) [33]. Thus, it can be concluded that exosomes regulate the occurrence of ferroptosis by affecting the level of lipid peroxidation.

### Amino acid metabolism

**Fig. 2:**
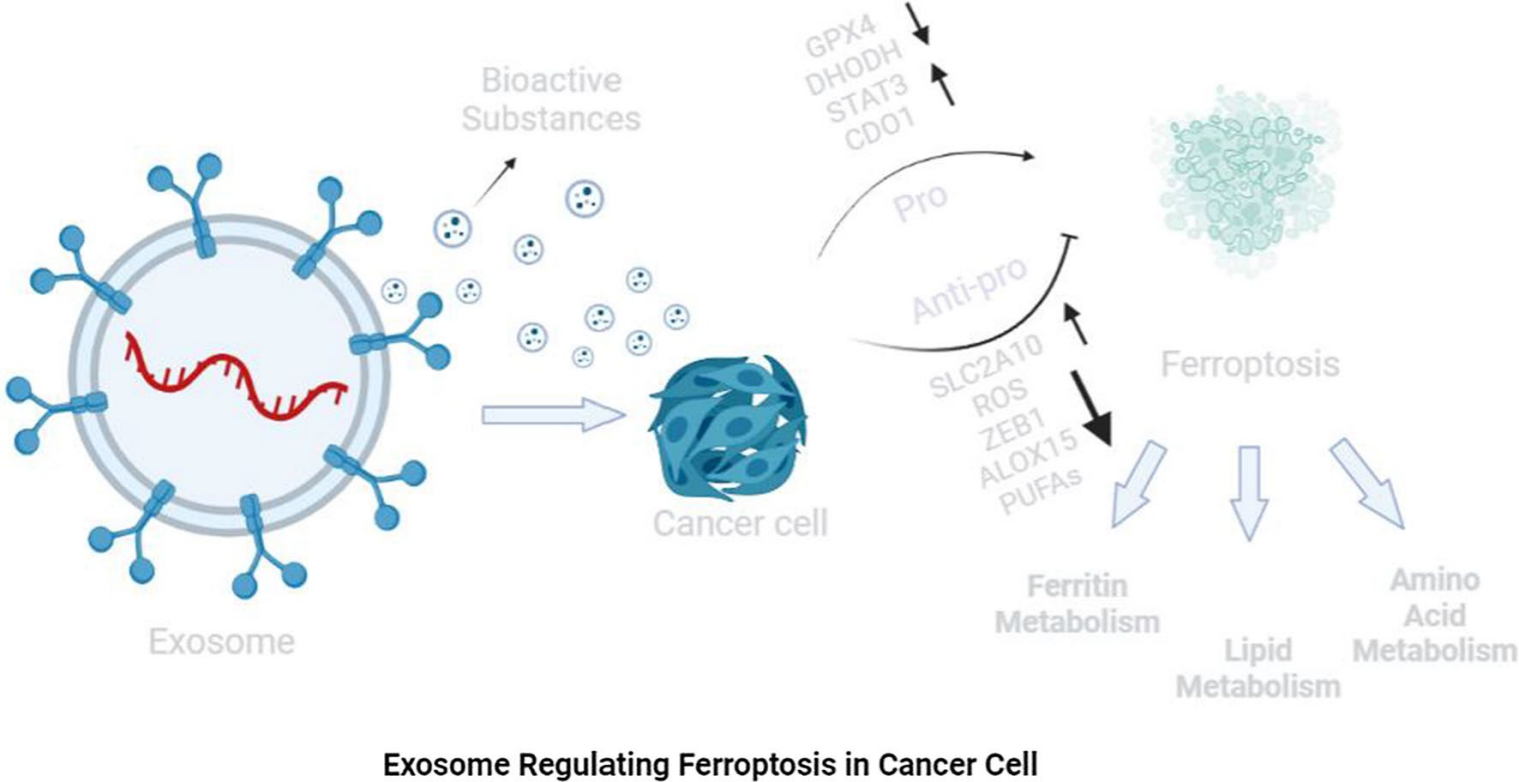
Exosomes regulate ferroptosis of cancer cells. Exosomes can regulate ferroptosis in three ways: ferritin metabolism, lipid metabolism and amino acid metabolism. Exosomes release bioactive substances to regulate the levels of GPX4, DHODH, STAT3, CDO1, SLC2A10, ROS, ZEB1, ALOX15, and PUFAs to affect ferroptosis of cancer cells

In vivo and in vitro experiments showed that treatment with MSCs and MSC-exos decreased the mRNA levels of prostaglandin-endoperoxide synthase 2 (PTGS2) and lipoxygenases in primary liver and mouse liver cells, thus restoring the expression of SLC7A11 protein; these results suggest that the protective effect of MSCs and MSC-exos on acute liver injury (ALI) is partially achieved by inhibition of ferroptosis [34]. Lin et al. further revealed that the expression of SLC7A11 protein induced by MSC-exos was accompanied by an increase in CD44 and OTUB1 expression. OTUB1-mediated deubiquitination can rescue the abnormal level of ubiquitinated SLC7A11 induced by carbon tetrachloride (CCI_4_), thus enhancing the stability of SLC7A11 to contribute to the activation of system XC- and inhibiting CCI_4_-induced ferroptosis in hepatocytes [34]. These findings provide new evidence that exosomes participate in ferroptosis pathways related to antioxidant system balance and can play a role in the treatment of diseases. In another study of dexamethasone and endothelial progenitor cell-derived EVs (EPC-EVs) in the treatment of steroid-induced osteoporosis in mice, Kyoto Encyclopedia of Genes and Genomes (KEGG) pathway enrichment analysis found that the levels of GPX4 and system XC- in mouse osteoblasts were decreased after high-dose dexamethasone treatment, which activated ferroptosis. On the other hand, dexamethasone-induced ferroptosis of mouse osteoblasts could be reversed by adding EPC-EVs [35]. A subsequent study of the regulatory effect of exosomes derived from M2 macrophages on ferroptosis in laryngeal cancer showed that exosomes derived from M2 macrophages induced upregulated expression of GPX4 and glutathione in erastin-treated TU212 cells and decreased the levels of intracellular ROS and malondialdehyde, thus inhibiting ferroptosis in cancer cells [36]. In summary, these studies have demonstrated that exosomes can regulate ferroptosis by regulating key mediators and metabolites in the amino acid metabolism pathway. The above results further suggest that exosomes not only participate in ferritin metabolism and lipid metabolism pathways in ferroptosis but also participate in amino acid metabolism pathways and play a role in regulating ferroptosis (Fig. 2).

## Exosome-induced regulation of ferroptosis in malignant tumor treatment

Malignant cancers have some of the highest mortality rates among diseases [37]. At present, the effectiveness of surgery, chemotherapy, targeted therapy and immunotherapy in cancer is still limited. Therefore, it is particularly urgent to explore other strategies to induce cancer-specific cell death. A large number of studies have proven that exosomes, as an important mediator of intercellular communication, inhibit tumor invasion and metastasis by inducing ferroptosis of cancer cells, but a further understanding of the pathological mechanism of exosomes in tumorigenesis is needed to find new methods and approaches for targeted therapy of malignant tumors [32]. However, exosomes can also negatively regulate ferroptosis in diseases, and thus, exosomes must be applied in reasonable and standardized strategies to appropriately regulate ferroptosis [38].

### Exosomes promote ferroptosis in malignant tumors

One study has shown that knockout of prominin-2 expression in exosomes can restore the iron concentration in breast cancer cells, reduce iron efflux, inhibit the secretion of tumor-derived exosomes, and thus enhance ferroptosis in breast cancer cells [39].

An in vivo and in vitro experiment showed that a hepatocellular carcinoma (HCC)-targeted exosome system (Exo^SP94-Lamp2b-RRM^) could specifically transfer multiple siRNAs to HCC tissue and enhance the ferroptosis induced by sorafenib by silencing the expression of GPX4 and DHODH, thus increasing the sensitivity of HCC cells to sorafenib. These results provided a new way to overcome sorafenib resistance in the clinic [40]. In tissue samples of patients with non-small-cell lung carcinoma (NSCLC) responsive to cisplatin, it was found that the level of exosomal miR-4443 in cisplatin-resistant NSCLC tissue-derived exosomes was upregulated compared with that in cisplatin-sensitive NSCLC tissue-derived exosomes, and overexpressed exosomal miR-4443 negatively regulated the modification of human fibroblast-specific protein 1 (FSP1) m6A induced by methyltransferase-like protein 3 (METTL3) in A549-S cells and mediated ferroptosis to promote drug resistance in NSCLC [41]. These results show that exosomal miR-4443 is an important target regulatory gene that can affect the levels of exosomes and promote cancer cell ferroptosis to inhibit cancer cell proliferation and metastasis and reduce chemotherapy resistance.

Exosomes derived from tumor cells can inhibit the function of dendritic cells (DCs) and T cells. Excessive secretion of programmed death ligand 1 (PD-L1) leads to resistance to and clinical failure of programmed death 1 (PD-1)/PD-L1 immunotherapy. Application of anti-exosomal PD-L1 therapy can restore T cell function and enhance the ferroptosis of malignant melanoma (MM) cells to relieve inhibition of antitumor immunity [42]. Wang et al. developed nanoparticles carrying an amphiphilic hyaluronic acid-assembled exosome inhibitor (GW4869) and a ferroptosis inducer (Fe3+) that induced ferroptosis of B16F10 MM cells through synergistic effects of the two active components, enhanced the antitumor immune response to MM cells, stimulated cytotoxic T lymphocytes (CTLs) and immune memory, and prevented the metastasis of tumor cells throughout the body [43]. Hu et al. developed an exosome-like vesicle vaccine derived from tumor cells genetically engineered to express fibroblast activation protein (eNVs-FAP). The results showed that the cellular immune response activated by eNVs-FAP can promote tumor ferroptosis by inducing the release of interferon-gamma (IFN-γ) from CTLs and eliminating FAP^+^ cancer-associated fibroblasts (CAFs), which play a role in antitumor immunity [44]. These studies found that strengthening the inducing effect of exosomes on ferroptosis may improve antitumor immunity, providing a new targeted anticancer immunotherapy strategy. Studies have shown that KRAS is a key mediator of cancer cell-macrophage communication. Oxidative stress leads to the release of the KRAS^G12D^ protein from cancer cells by inducing autophagy-dependent ferroptosis. Extracellular KRAS^G12D^ can be loaded into exosomes and ingested by macrophages, resulting in macrophage oxidation through signal transducer and activator of transcription 3 (STAT3)-dependent fatty acid signaling. This causes the transformation of macrophages into an M2-like tumor-promoting phenotype and stimulates the growth of pancreatic ductal adenocarcinoma (PDAC) tumors induced by macrophages [45]. These finding suggest that preventing carcinogenic macrophages from ingesting exosomal KRAS^G12D^ protein plays an important role in inducing iron-related autophagy in cancer cells and regulating immune metabolism in the tumor microenvironment.

In glioblastoma (GBM), the poor drug permeability of the blood‒brain barrier (BBB) has long been a major obstacle to tumor treatment efficacy. Exosomes modified to carry angiopep-2 peptides can trigger cell penetration and not only have the ability to penetrate the BBB but also enhance ferroptosis by recognizing lipoprotein receptor protein 1 (LRP-1) receptors and targeting GBM cells [46]. An in vivo and in vitro experiment showed that engineered exosomes including CD47, the ferroptosis inducer erastin and the photosensitizer Rose Bengal (RB) induced significant ferroptosis in HCC, with the lowest toxicity seen in the liver and kidney, providing a treatment option for patients with HCC [47]. Other studies have shown that treatment with the ferroptosis inducer erastin combined with folate (FA)-secreting exosomes (erastin@FA-exos) inhibited the expression of intracellular GPX4, thus upregulating the expression of cysteine dioxygenase 1 (CDO1). In addition, erastin@FA-exos significantly increased glutathione consumption and the production of ROS in MDA-MB-231 cells and effectively induced ferroptosis of triple-negative breast cancer (TNBC) cells [38]. Therefore, this exosome-based targeted drug delivery system may be an innovative and powerful delivery platform for the treatment of cancer.

Contrary to many experimental results, Hu et al. found that induction of M1 macrophage ferroptosis with hepatitis B virus (HBV)-positive hepatoma cell-derived exosomal miR-142-3p promoted the progression of liver cancer [48].

The above results support that exosome-induced ferroptosis can affect tumor cell proliferation and metastasis and reverse the chemotherapy resistance of malignant tumors [38] (Table 1).

**Table 1:** Exosomes promote ferroptosis in malignant tumors

Type of tumor	Key findings	References
BC	Decreased levels of prominin-2 in exosomes restored iron concentration, reduced iron efflux, inhibited the secretion of tumor-derived exosomes, and increased ferroptosis of BC cells	[27]
HCC	Exo^SP94-Lamp2b-RRM^ enhanced the ferroptosis induced by sorafenib by silencing the expression of GPX4 and DHODH	[28]
NSCLC	Exosomal miR-4443 negatively regulated the modification of FSP1 m6A induced by METTL3 to mediate ferroptosis, thus promoting drug resistance of NSCLC	[29]
MM	Anti-exosomal PD-L1 restored T cell function and increased the ferroptosis of MM cells to restore antitumor immunity	[30]
MM	GW4869 and Fe3+ nanoparticles, which can induce ferroptosis of B16F10 MM cells, enhanced the antitumor immune response to MM cells, stimulated cytotoxic T lymphocytes and immune memory, and prevented MM metastasis	[31]
CRC LUAD MM BC	ENVs-FAP promoted tumor ferroptosis by inducing the release of IFN-γ from CTLs and consuming FAP^+^ CAFs, which played a role in antitumor immunity	[32]
PDAC	Extracellular KRAS^G12D^ was ingested by macrophages, resulting in macrophage oxidation through STAT3-dependent fatty acids. This caused the transformation of macrophages into an M2-like tumor-promoting phenotype and stimulated the growth of PDAC induced by macrophages	[33]
GBM	Exosomes modified by angiopep-2 peptides triggered cell penetration and not only penetrated the BBB but also increased ferroptosis by recognizing LRP-1 receptors and targeting GBM cells	[34]
HCC	Engineered exosomes composed of CD47, erastin and RB induced significant ferroptosis in HCC	[35]
TNBC	Erastin@FA-exo treatment inhibited the expression of intracellular GPX4, upregulated the expression of CDO1, significantly increased glutathione consumption and the production of ROS in MDA-MB-231 cells, and effectively induced ferroptosis in TNBC	[26]
HCC	HBV-positive hepatoma cell-derived exosomal miR-142-3p induced ferroptosis of M1 macrophages, promoting the progression of HCC	[36]

*BC* breast cancer, *HCC* hepatocellular carcinoma, *GPX4* glutathione peroxidase 4, *DHODH* dihydroorotate dehydrogenase, *NSCLC* non-small-cell lung carcinoma, *FSP1* fibroblast-specific protein 1, *METTL3* methyltransferase-like protein 3, *PD-L1* programmed death ligand 1, *MM* malignant melanoma, *CRC* colorectal cancer, *LUAD* lung adenocarcinoma, *IFN-γ* interferon-gamma, *CTLs* cytotoxic T lymphocytes, *PDAC* pancreatic ductal adenocarcinoma, *GBM* glioblastoma, *BBB* blood–brain barrier, *LRP-1* lipoprotein receptor protein 1, *RB* rose Bengal, *TNBC* triple-negative breast cancer, *CDO1* cysteine dioxygenase 1, *ROS* reactive oxygen species

### Exosomes inhibit ferroptosis in malignant tumors

A study found that prominin-2 is a ferroptosis stress response protein that promotes the formation of ferritin-containing MVB and exosomes, which transport iron out of cells, thereby inhibiting ferroptosis in breast epithelium and breast cancer cell ferroptosis. These effects block ferritin output mediated by MVBs and exosomes, inhibiting GPX4 and resulting in intracellular iron accumulation [29]. A bioinformatics study showed that LINC02381-encoding micropeptides inhibit ferroptosis in GBM by upregulating the glucose transporter SLC2A10 [49]. Studies of the regulatory effect of exosomes derived from M2 macrophages on ferroptosis in laryngeal cancer showed that such exosomes induced upregulated expression of GPX4 and glutathione in erastin-treated TU212 cells and downregulated the levels of intracellular ROS and malondialdehyde, thus inhibiting the ferroptosis of cancer cells [36].

In an in vivo and in vitro experiment, Zhang et al. found that exosomes derived from hypoxia-induced NSCLC directly transmitted radioresistance to surrounding NSCLC cells with normal oxygen content in a manner dependent on exosomal angiopoietin-like 4 (ANGPTL4) and GPX4, thus inhibiting the occurrence of ferroptosis and leading to tumor progression. These innovative findings regarding the relationships between ANGPTL4, ferroptosis and hypoxia-induced radioresistance reveal a role of intracellular ANGPTL4 and hypoxia-induced exosomal ANGPTL4 in driving radioresistance and the potential of exploiting these relationships to improve the clinical outcome of NSCLC [50]. Subsequently, with a mouse model of lung adenocarcinoma (LUAD) and tissue samples from patients with LUAD, researchers demonstrated that exosomal RNA‒protein interactions can regulate cancer cell ferroptosis. This regulation may be due to the interaction between exosomal cir93 and fatty acid-binding protein 3 (FABP3). FABP3 transports arachidonic acid (AA) and promotes its reaction with taurine, resulting in a decrease in global AA and decreased lipid peroxidation, which desensitizes LUAD cells to ferroptosis. Therefore, exosomal cir93 is very important for desensitizing LUAD cells to ferroptosis. Reducing exosome-related desensitization to ferroptosis may be helpful for the treatment of LUAD [33].

It is well known that obesity is closely related to the poor prognosis of patients with advanced colorectal cancer (CRC). Zhang et al. found that increased microsomal triglyceride transfer protein (MTTP) in adipocyte-derived exosomes reduced the sensitivity of CRC cells to ferroptosis, thus promoting resistance of the cells to oxaliplatin. Further mechanistic analysis showed that the MTTP/proline-rich acidic protein 1 (PRAP1) complex inhibits the expression of zinc-finger E-box binding homeobox 1 (ZEB1) and increases the levels of GPX4 and cystine. This leads to a decrease in the proportion of PUFAs and the level of lipid ROS. In addition, inhibition of MTTP has been shown to increase the sensitivity of cells to chemotherapy in obese tumor transplantation model mice [51]. miR-522 secreted from CAF-exos inhibits the expression of ALOX15 and the accumulation of lipid ROS, inhibiting the ferroptosis of GC cells and resulting in chemoresistance in GC [32].

Macrophage migration inhibitory factor (MIF) is highly expressed in nasopharyngeal carcinoma (NPC) cells, and MIF secreted from exosomes can be absorbed by macrophages, thus inhibiting ferroptosis of macrophages and promoting NPC metastasis. Targeting MIF may be a strategy to reduce the metastasis rate [52].

It has been shown that exosomal lnc-ENDOG-1:1 (lncFERO) from GC cells directly interacts with stearoyl-CoA-desaturase 1 (SCD1) mRNA to promote the expression of SCD1 by aggregating heterogeneous nuclear ribonucleoprotein A1 (hnRNPA1), resulting in downregulation of PUFAs and inhibition of GC stem cell ferroptosis. Targeting exosome-mediated ferroptosis of GC stem cells may be an effective method to prevent chemotherapy resistance and GC recurrence [53].

In summary, exosomes play a role in regulating cell proliferation, the immune response and the tumor microenvironment by promoting or inhibiting ferroptosis of cancer cells, and these ideas improve the understanding and provide an experimental basis for tumor chemotherapy resistance. However, at present, the role of tumor cell ferroptosis induced by the combined action of proteins and lipids carried by exosomes in pathological processes such as anti-angiogenic and inflammatory reactions has not been clearly described, and strategies that aim to regulate ferroptosis via exosomes in tumors have not been extensively applied. More related studies are needed to provide new ideas for tumor treatment (Table 2).

**Table 2:** Exosomes inhibit ferroptosis in malignant tumors

Type of tumor	Key findings	References
BC	Prominin-2 promoted the formation of ferritin-containing MVBs and exosomes, which transported iron out of cells, inhibiting ferroptosis in BC, blocking ferritin output mediated by MVBs and exosomes, and inhibiting GPX4 expression, resulting in intracellular iron accumulation	[17]
GBM	LINC02381-encoding micropeptides inhibited ferroptosis in GBM by upregulating the glucose transporter SLC2A10	[37]
LSCC	Exosomes derived from M2 macrophages increased the levels of GPX4 and glutathione in erastin-treated TU212 cells and decreased the levels of intracellular ROS and malondialdehyde, inhibiting ferroptosis in LSCC	[24]
NSCLC	Exosomes derived from NSCLC induced by hypoxia directly transmitted radioresistance to the surrounding NSCLC cells with normal oxygen content in a manner dependent on exosomal ANGPTL4 and GPX4, inhibiting ferroptosis and leading to tumor progression	[38]
LUAD	Exosomal RNA−protein interactions regulated cancer cell ferroptosis. This regulation may be due to the interaction between exosomal cir93 and FABP3, which transports AA and promotes its reaction with taurine, resulting in a decrease in global AA and a reduction in lipid peroxidation and desensitizing LUAD cells to ferroptosis	[21]
CRC	The MTTP/PRAP1 complex in exosomes inhibited the expression of ZEB1, increased the levels of GPX4 and cystine, decreased of the proportion of PUFAs and the level of lipid ROS, and decreased the level of exosomal MTTP to increase the sensitivity of cells to chemotherapy in obese tumor transplantation model mice	[39]
GC	miR-522 from CAF-exos decreased the expression of ALOX15 and the accumulation of lipid ROS, inhibiting the ferroptosis of GC cells and resulting in chemoresistance in GC	[20]
NPC	Exosomal MIF was absorbed by macrophages, which inhibited the ferroptosis of macrophages and promoted the metastasis of NPC	[40]
GC	Exosomal lncFERO from GC cells directly interacted with SCD1 mRNA to promote the expression of SCD1 by aggregating hnRNPA1, resulting in decreased levels of PUFAs and inhibition of GC stem cell ferroptosis	[41]

*BC* breast cancer, *MVBs* multivesicular bodies, *GPX4* glutathione peroxidase 4, *GBM* glioblastoma, *LSCC* laryngeal squamous cell carcinoma, *ROS* reactive oxygen species, *NSCLC* non-small-cell lung carcinoma, *ANGPTL4* angiopoietin-like 4, *FABP3* fatty acid-binding protein 3, *LUAD* lung adenocarcinoma, *AA* arachidonic acid, *MTTP* microsomal triglyceride transfer protein, *PRAP1* proline-rich acidic protein 1, *ZEB1* zinc-finger E-box binding homeobox 1, *PUFAs* polyunsaturated fatty acids, *CRC* colorectal cancer, *GC* gastric cancer, *CAF-exos* cancer-associated fibroblast-derived exosomes, *ALOX15* arachidonate lipoxygenase 15, *NPC* nasopharyngeal carcinoma, *MIF* migration inhibitory factor, *SCD1* stearoyl-CoA-desaturase 1

## Conclusions and perspectives

This paper focuses on the regulation of ferroptosis by exosomes and discusses strategies that employ exosomes to regulate ferroptosis as tumor treatment. Although the relationship between exosomes and ferroptosis remains controversial and there many research factors remain to be standardized, such as exosome system preparation and the source of exosomes, the relationship between ferroptosis and exosomes is a novel research direction. Relevant studies will provide a reference for improving the therapeutic effect in various diseases and drug research and development.

Until now, the potential roles of signal pathways relevant to ferroptosis in cancer had been reported, including those related to p53, noncoding RNA, and the tumor microenvironment and so on in other study. Along with the above mechanism we summarized, we believe some ferroptosis-based cancer therapies, such as clinical drugs, nanomaterials, exosomes and gene technology will be applied in clinical cancer treatment to benefit more patients to achieve better outcomes.
